# The evolutionary loss of the Eh1 motif in FoxE1 in the lineage of placental mammals

**DOI:** 10.1371/journal.pone.0296176

**Published:** 2023-12-27

**Authors:** Mahak Sharma, Victoria M. Larow, Nataliia Dobychina, Daniel S. Kessler, Maria M. Krasilnikova, Sergey Yaklichkin

**Affiliations:** 1 Department of Biochemistry and Molecular Biology, Penn State University, University Park, Pennsylvania, United States of America; 2 Cancer Biology and Genetics Program, Memorial Sloan Kettering Cancer Center, New York, New York, United States of America; 3 Department of Cell and Developmental Biology, Perelman School of Medicine, University of Pennsylvania, Philadelphia, Pennsylvania, United States of America; University of Colorado Boulder, UNITED STATES

## Abstract

Forkhead box E1 (FoxE1) protein is a transcriptional regulator known to play a major role in the development of the thyroid gland. By performing sequence alignments, we detected a deletion in FoxE1, which occurred in the evolution of mammals, near the point of divergence of placental mammals. This deletion led to the loss of the majority of the Eh1 motif, which was important for interactions with transcriptional corepressors. To investigate a potential mechanism for this deletion, we analyzed replication through the deletion area in mammalian cells with two-dimensional gel electrophoresis, and in vitro, using a primer extension reaction. We demonstrated that the area of the deletion presented an obstacle for replication in both assays. The exact position of polymerization arrest in primer extension indicated that it was most likely caused by a quadruplex DNA structure. The quadruplex structure hypothesis is also consistent with the exact borders of the deletion. The exact roles of these evolutionary changes in FoxE1 family proteins are still to be determined.

## Materials and methods

### DNA and protein sequences and their analysis

Protein and DNA sequences were assembled and analyzed using the software Accelerus gene 2.5 (Accelerus Inc.), and the software Bio4Life 1.0.0.1 (D. Boyko and S. Yaklichkin, unpublished). Analysis of secondary DNA structure of *FoxE1* genes was performed with the Mfold webserver (dinamelt.bioinfo.rpi.edu/zipfold.php). FoxE1 protein and genomic sequences were obtained from the NCBI database (ncbi.nlm.nih.gov) and the Ensemble Genome Browser 47 server (ensembl.org). Single-exon *FoxE1* genes from fish, *Gasterosteus aculeatus* (ENSGACE00000205237) and *Oryzias latipe*s (ENSORLP00000008910), contained some errors, which were corrected; uninterrupted ORFs were predicted.

The *FoxE1* gene of *Sus scrofa* was assembled using the contig of the clone CH242-205E18. The accession numbers of *FoxE1* sequences obtained from the Ensemble server were as follows: *Loxodonta Africana (*ENSLAFP00000015306), *Vicugna pacos (*ENSVPAP00000009810), *Dasypus novemcinctus* (ENSDNOP00000006203), *Pongo pygmaeus (*ENSPPYP00000021771), and *Xenopus tropicalis (*FGENESH00000105173). The analyzed sequences from the NCBI database were *FoxE1* from *Ornithorhynchus anatinus* (XP_001517796), *Monodelphis domestica* (XP_001372714), *Homo sapiens* (NP_004464), and *Mus musculus* (NP_899121.1). Identification of Eh1 motifs in FoxE1 protein sequences was performed in accordance with the previously described methods of sequence analysis [1].

### Sequencing of *FoxE1* genes

Genomic DNA samples of *Macropus eugenii* (Tammar wallaby) and *Tachyglossus aculeatus* (echidna) were kindly provided by Dr. Ke-Jun Wei (Comparative Genomics Group, Research School of Biological Sciences, Australian National University). PCR fragments of genomic DNA of *FoxE1* of *Macropus eugenii* were generated using a set of PCR primers selected using conserved regions of the *FoxE1* gene of *Monodelphis domestica*. A partial sequence of the *FoxE1* gene of *Tachyglossus aculeatus* was determined by generating a set of overlapping PCR fragments using sets of PCR primers. PCR primers were designed to conserved regions, shared between the *FoxE1* genes of *Ornithorhynchus anatinus* and *Monodelphis domestica*. In addition, a NotI fragment of the *FoxE1* gene of *Tachyglossus aculeatus* was subcloned into the pCRII vector (Invitrogen) and sequenced with standard primers. Sequences of *FoxE1* genes of *Macropus eugenii* and *Tachyglossus aculeatus* were deposited in the NCBI database under the accession numbers HM991738.1 and HQ111427.1.

The genomic DNA of *Elephantulus rufescens* was isolated from the blood of *Elephantulus rufescens* using a genomic DNA isolation kit (Qiagene). Blood of *Elephantulus rufescens* was kindly donated by the Philadelphia Zoo (philadelphiazoo.org). To amplify *FoxE1* of *Elephantulus rufescens*, primers were designed based on conserved regions shared between the *FoxE1* genes of *Homo sapiens*, *Mus musculus* and *Loxodonta africana*. PCR fragments obtained were sequenced by the automated Sanger method. *FoxE1* gene sequences were assembled using software Accelrus gene 2.5 and Bio4Life 1.0.0.1. All designed primer sequences are available by request.

### Sequence alignments and phylogenic analysis of Fox proteins

A phylogenic tree for the FoxE subclass was generated based on the forkhead DNA-binding domain sequences (100 residues) for FoxC, D, H, Q, E subclasses. Multiple sequence alignments were constructed using the software Clustal W [2] and T-COFFEE, version 7.7.1 (tcoffee.vital-it.ch/cgi-bin/Tcoffee/tcoffee_cgi/index.cgi) [3], and these sequences were converted into a cladogram using the software MEGA 3.1 (metameme.sdsc.edu). Sequence distances were calculated with Poisson correction, and a neighbor-joining method was used to construct the tree topology with bootstrap analysis of 1000 samples.

### Plasmids and transfections

PucneoFox and PucneoFoxRev plasmids were obtained by cloning the hairpin-forming region of the echidna *FoxE1* gene into the PucNeo plasmid, designed for DNA replication analysis in mammalian cells. PucNeo was obtained from pSV2neo plasmid (Stratagene) by inverting the region containing the ColE1 origin and ampicillin resistance gene [4]. The sequence, corresponding to the hairpin-forming region of the echidna *FoxE1* gene, accession number HQ111427.1 (------), was first inserted into the blunt-ended *Eco*81I-site of the pYES-Bsg plasmid [5], followed by subcloning of *FoxE1*-containing HindIII fragment in two orientations into the blunt-ended AatII site of Pucneo. All cloning and DNA isolation steps were performed in *E*. *coli* XL1-blue strain (Stratagene). COS-1 monkey fibroblasts (ATCC CRL1650TM) were transfected with 5 μg of plasmid DNA using Lipofectin reagent (Invitrogen), according to manufacturer’s instructions.

### Analysis of single-stranded regions by chloroacetaldehyde modifications and primer extension reactions

PucneoFox and PucneoFoxRev plasmids containing the Fox region in two orientations were incubated in 100 mM KCl, 10 mM NaCl, 0.5 mM MgCl, and 20 mM Tris-HCl, pH 7, to mimic the intracellular environment. Each sample was then divided in two halves; one half was incubated with 0.5% chloroacetaldehyde at 37°C for 8 minutes; while the second half was used as a control, which underwent the same protocol, except for the addition of chloroacetaldehyde. Chloroacetaldehyde modifies mostly single-stranded As and Cs [6]. Reactions were stopped by the addition of 5 volumes of prechilled 0.3 M NaOAc pH 7, followed by two rounds of ethanol precipitation, and two washes with 70% ethanol. The control and chloroacetaldehyde samples were then linearized by ScaI-HF restriction endonuclease, followed by another round of precipitation, and finally dissolved in water, and subjected to 60% formic acid treatment for 5 min at room temperature to convert chloroacetaldehyde-modified sites into single-stranded breaks. The reactions were stopped by addition of 5 volumes of prechilled 0.3 M NaOAc pH 7, followed by ethanol precipitation and 70% ethanol wash. Upon drying, the samples were dissolved in TE buffer (10mM Tris-HCl, pH 8.0, 1mM EDTA), and heated at 65°C for 10 min to minimize secondary structures. Next, primer extension reactions were performed with Taq polymerase (NEB) in a standard buffer with 2 mM Mg^2+^, and only a forward (no reverse) FWD primer, 5’-CAAATAGGGGTTCCCCCCACATTTCC-3’, that annealed about 100 nt from the start of the cloned *FoxE1* region. The primer extension reactions were performed in a thermo-cycler using the following cycling conditions: initial denaturing at 95°C for 30 sec, then 60 cycles of denaturing 95°C for 10 sec, annealing 58°C for 30 sec, and extension at 72°C for 30 seconds. Control primer extension reactions were performed using maxiprep samples, linearized with ScaI-HF, precipitated, and heated in TE, but not preincubated with ions, or subjected to chloroacetaldehyde and formic acid. The length controls were obtained with FWD and R1 5’-ATCGAATTTGAGGTCTGCACTCGAG-3’, and FWD and R2 5’-GTTCAGCCTCAACGGGCT-3’ primers combinations. R1 annealed right before the GC-rich area, and R2 annealed at the very end of *FoxE1* region (Fig 2A). The reactions and controls were mixed with the same volumes of loading dye (95% formamide, 0.025% (w/v) Bromophenol blue, 0.025% (w/v) Xylene cyanol, 5 mM EDTA pH 8.0), denatured for 3 min at 70°C, and separated in 6% denaturing polyacrylamide gel with urea. The sequencing reactions were performed with Thermo Sequenase™ Cycle Sequencing Kit (ThermoFisher Scientific)), using the same forward primer as in primer extension reactions, according to manufacturer’s instructions. Afterwards, the gel was electro-transferred onto a Nytran Supercharge blotting membrane (Whatman). The membrane was hybridized with a biotinylated probe, obtained by PCR with Biotin-11 dUTP, using *Fox*-containing plasmid as a template, the same forward primer as used in primer extension, and the reverse R1 primer that annealed at the position immediately upstream from the cloned *FoxE1* region. Hybridization was performed overnight in Church buffer, followed by two 10 min washes in 0.1xSSC, 0.1% SDS, at room temperature, and at 60°C. Lastly, the detection was carried out by blocking the membrane in Blocking buffer (0.25 M Na_2_HPO_4_, pH 7.2, 5% (w/v) SDS), followed by incubation with streptavidin alkaline phosphatase conjugate dissolved in Blocking buffer, with subsequent steps using Blocking/Washing and Detection buffers from Biotin Chromogenic Detection kit (ThermoFisher Scientific), according to the supplier’s recommended protocol. Then, the fluorescent detection was performed with CDP-star reagent (Cytiva Amersham), according to the instructions provided by manufacturer. The membrane was then exposed with X-Ray film and the bands were interpreted by comparison to the sequencing lanes.

### Isolation of replication intermediates and 2D gel electrophoresis

COS-1 cells were transfected with PucNeoFox and PucNeoFoxRev plasmids, which contained the echidna *FoxE1* region in both orientations relative to the replication origin. The plasmid PucNeo, which did not contain the *FoxE1* region, was used in a control transfection. Intermediate products of replication of the plasmids were isolated from COS-1 cells, digested with DpnI and AflIII enzymes, and separated by neutral-neutral two-dimensional agarose gel electrophoresis. DpnI digest was used to eliminate parental plasmid molecules, which did not go through at least two rounds of DNA replication in mammalian cells. Isolation of replication intermediates was performed using the modified Hirt’s extraction protocol [7]. Cells were washed with 5 ml of 50 mM Tris-HCl pH 7.0, 150 mM NaCl, and lysed in 0.75 ml of lysis solution (50 mM Tris-HCl, 20 mM EDTA, 10 mM NaCl, 10% SDS, 10 μl of 20 mg/ml of Proteinase K (Promega)) for 15–25 minutes at room temperature. The lysates were scraped off the plate, incubated for 8–24 hours in the presence of 1M NaCl, and cleared by centrifugation at 27,000 g at 4°C for 50 minutes. Supernatants were incubated with 5 μl of 20 mg/ml Proteinase K at 55°C for 2 hours, followed by phenol/chloroform, and chloroform extraction. Replication intermediates were precipitated with 1 volume of isopropanol, washed with 70% ethanol, and dissolved in TE buffer. Replication intermediates, digested with restriction enzymes, were separated in the first dimension in 0.4% agarose gel at 1 V/cm for 13 hours, followed by separation in the second dimension in 1% agarose at 5 V/cm for 8 hours at 4°C as described [7,8]. The gel was transferred to a Hybond XL nylon membrane, and hybridized with a probe shown in Fig 3A. Quantitative analysis of replication intermediates was conducted using the Molecular Dynamics Phosphoimager as described [8].

## Introduction

The Fox gene family is a large and functionally diverse family of forkhead-related transcriptional regulators, which are essential for embryogenesis and adult physiology. The majority of Fox family proteins contain an engrailed homology 1 (Eh1) motif positioned C-terminal to the forkhead-related DNA-binding domain [1]. The Eh1 motif is a short eight amino acid sequence, FS(I/V)XXΦΦX, where Φ is a branched hydrophobic residue. Eh1 provides a specific binding surface on Fox proteins for recruitment of the Groucho/TLE protein co-repressor complex. The physical interaction of the Eh1 motif with Groucho/TLE co-repressors was shown for a variety of Fox family members, such as FoxA2, FoxG1, Slp2 (FoxG sub-family), FoxD3, and FoxH1 [1,9–13]. The Fox-Groucho/TLE complex can repress transcription of target genes either directly, or through reducing transactivation mediated by co-activators [9]. Moreover, the recruitment of Groucho/TLE by FoxA2 generates a compact chromatin structure resulting in repression of target genes [14].

While the Eh1 motif is present in most members of the Fox family proteins, for individual Fox family genes, it was lost during evolution in certain species. In our previous study, we reported the absence of the Eh1 motif in some mammalian FoxE1 proteins [1]. *FoxE1* is a single-exon gene encoding a transcriptional regulator, required for the development of the thyroid gland, the palate, and for the hair morphogenesis [15]. FoxE1 has been shown to be functionally indispensable in mice; FoxE1 null mice exhibit a cleft palate, and either a sublingual or a completely absent thyroid gland [15]. Mutations in the human *FoxE1* gene lead to Bamforth syndrome, which is characterized by thyroid agenesis, cleft palate, spiky hair, and choanal atresia [16,17].

In general, mutational events correlate with formation of non-B DNA structures, such as hairpins, triplexes, quadruplexes, Z DNA, slipped strand DNA, and i-motifs [18–26]. Formation of non-B DNA can transiently occur during the processes of replication and transcription, or can exist more stably in genomes throughout the cell cycle [23]. Alternative DNA conformations, including hairpin, triplex, and G-quadruplex structures, were also shown to affect replication elongation [5,27–30].

Replication stalling has been demonstrated at trinucleotide repeats, such as (GAA)_n_, (CGG)_n_, and (CTG)_n_, which expand in trinucleotide repeats diseases. All these repeats have a potential to form unusual DNA conformations (reviewed in [20]. For example, GAA repeats can form triplexes, while CTG and CGG repeats mostly form hairpins [19]. The formations of these structures in the living cells have been directly demonstrated by multiple methods [31]. Formation of quadruplexes has been revealed at stalled replication forks [32]. Truncated DNA strands, resulting from replication stalling, can invade the neighboring DNA regions, triggering DNA repair. Alternatively, they can cause the fork reversal [33], resulting in a double-stranded DNA end, which can also initiate recombination. Repair and recombination can result in healing of the DNA damage. However, such healing increases the risk of DNA changes, such as deletions, duplications, or sequence rearrangements [20]. Chromosomal fragility was observed at the GGC, AT, and other repeats that can form alternative DNA conformations in yeast [28,30,34].

Sequences that can form non-B structures are hot spots for genomic instability [35–37], associated with inherited disorders and cancer [20]. They can also play a significant role in evolution [23,38]. A study of microsatellite repeats in primate evolution showed increased mutagenicity associated with repeats having non-B DNA structure potential [39]. An analysis of human-orangutan divergence showed an elevation in substitution frequencies corresponding to the single-stranded regions of non-B DNA structures [23].

Many sequences, especially those that are G-rich, have a potential to fold into several types of alternative conformations. DNA structure of the same sequence can differ in different cell types, and even within the same cell. For example, H-DNA mostly forms during S-phase, and is cell-type specific. It has been shown to occur more frequently in transformed cell lines and differentiating cells, compared to iPSCs. The formation of non-B DNA structures can be affected by cellular processes, such as transcription and replication, since they can create temporarily single-stranded DNA regions that facilitate formation of alternative DNA structures [40].

In this study, we examined the timing of the loss of the Eh1 motif from the C-terminal domain of FoxE1 proteins. We have also investigated a potential molecular mechanism of this mutation and concluded that this loss likely occurred in a common ancestor of mammals due to the formation of a noncanonical DNA structure in the Eh1 region during DNA replication. We hypothesized that this deletion resulted in a loss of a co-repressor recruitment function, and possibly a gain of a novel function in transcriptional regulation by FoxE1.

## Results

### Loss of the Eh1 motif in FoxE1 proteins of placental mammals

In our previous study, we identified a loss of the Eh1 motif in a subset of FoxE1 proteins of placental mammals [1]. Here, we explore whether the Eh1 motif was lost in an ancestor of all mammals or later, in the evolution of placentals. To this end, the FoxE1 proteins of selected monotreme and marsupial mammals that diverged from placentals, were sequenced and examined for the presence of the Eh1 motif. Initial sequence analysis, performed in a previous study [1], identified the Eh1 motif in the FoxE1 protein sequences of the marsupial mammal *Monodelphis domestica* (opossum), and the monotreme mammal *Ornithorhynchus anatinus* (platypus). To confirm the presence of the Eh1 motif in FoxE1 proteins of other monotreme and marsupial mammals, FoxE1 of two additional species from both lineages, *Tachyglossus aculeatus* (echidna, monotreme*)*, and *Macropus eugenii* (kangaroo, marsupial), were sequenced. The presence of the Eh1 motif in FoxE1 proteins of those marsupial and monotreme species indicated that the loss of the Eh1 motif occurred later, in the lineage of placental mammals (Table 1).

**Table 1:** FoxE1 orthologs of monotreme and marsupial mammals contain an Engrailed Homology motif 1.

Protein	Species	Sequence	Accession number
FoxE1	*Ornithorhynchus anatinus*	FSLNGLMH	XP001217796
FoxE1	*Tachyglossus aculeatus*	FSLNGLVA	HQ111427
FoxE1	*Monodelphis domestica*	FSINSLVH	XP001372714
FoxE1	*Macropus eugenii*	FSINSLVH	HM991738
FoxD3	*Xenopus laevis*	FSIENIIG^a^	CAC12963
Gsc	*Homo sapiens*	FSIDNILA^b^	NP776248

^a^ The indicated sequence of FoxD3 binds to the XGrgr4 transcriptional co-repressor [12].

^b^ The indicated sequence of a Gsc homeodomain protein binds to the WD40 domain of the TLE1 transcriptional co-repressor [41].

**Fig 1:**
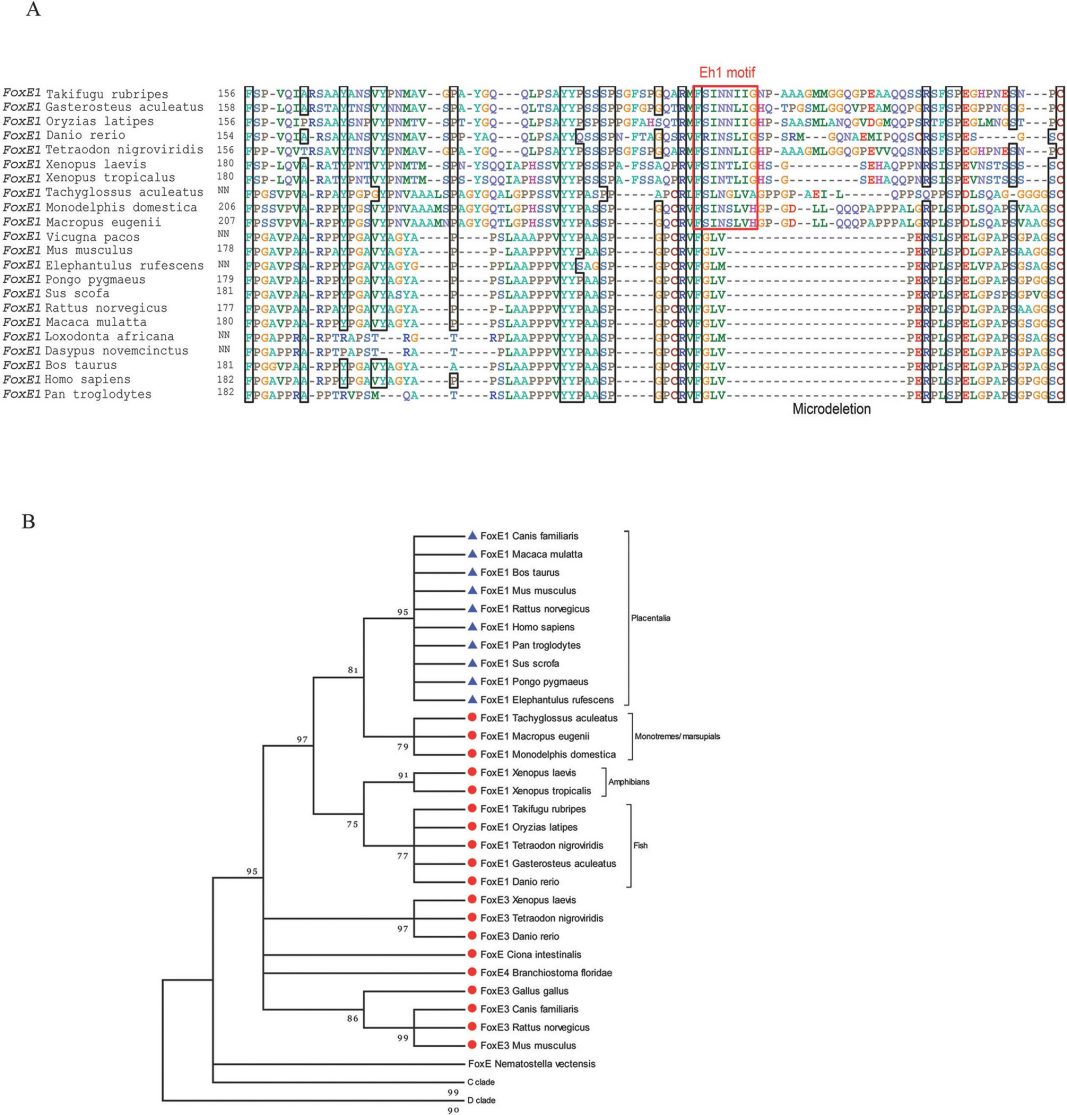
The C-terminal domain of FoxE1 of placental mammals contains a microdeletion of the Eh1 motif and adjacent region, which resulted in the loss of the Eh1 motif. (A) A multiple sequence alignment of the C-terminal domain of the FoxE1 proteins of vertebrates. (B) A phylogenetic tree for proteins of the FoxE subclass, and the FoxC and FoxD outgroups. A neighbor-joining method was used to construct the tree topology and bootstrapping values are shown at each branch point (percentage of 1000 bootstrap samples) using MEGA 3.1. The distance scale below the tree represents the number of substitutions per site. The C and D Fox outgroups have been collapsed for better illustration. Protein sequences that lack an Eh1 motif are represented by blue triangles. Fox proteins and subclasses that contain an Eh1 motif are represented by red circles.

To determine the evolutionary timing of the Eh1 deletion in placental mammals, we performed an alignment of the C-terminal regions of 22 FoxE1 proteins, 12 of which were derived from species from different orders of placental mammals, such as Rodentia, Afrotherians, Xenarthra, Proboscidea, Primates, and Artiodactyla. The FoxE1 sequences were aligned, and the accuracy was scored using the T-coffee algorithm [3] (Figs 1A and S1). The analysis demonstrated the loss of the Eh1 motif in all 12 FoxE1 proteins of placental mammals from our dataset, while confirming its evolutionary conservation in cold-blooded vertebrates and non-placental mammals.

To trace the evolution of Eh1 motif in FoxE proteins in metazoans, a phylogenetic tree of the FoxE subclass, along with the FoxC and FoxD outgroups, was constructed using a neighbor-joining method (Fig 1B). The topology of the phylogenetic tree indicated a close relatedness of all FoxE1 proteins examined, and a common ancestry of this subgroup. It also suggested that FoxE1 proteins from non-placental and placental mammals originated from a common ancestor, which contained the Eh1 motif.

Based on our phylogenetic analysis, we can estimate the timing of the deletion. Prior to the diversification of the placentals, the Eh1 motif was present in the FoxE1 proteins in non-placental mammals. The species we examined that split after placental divergence early in evolution forming the afrotheria clade, such as *Elephantulus rufescens* (elephant shrew), *Loxodonta africana* (African elephant), and *Dasypus novemcinctus* (nine-banded armadillo) did not include this motif. Thus, we concluded that the deletion already existed in the early evolution of the placentals.

Comparison of the Eh1 deletion sites across the placental mammals revealed that each had the same deletion borders. The resultant sequence lost four out of seven conserved residues of the Eh1 motif, along with 19 adjacent residues from the C-terminal side, while the other three conserved residues of the Eh1 motif remained in all of the analyzed placental mammalian sequences (in human FoxE1, residues 225–227, FGL). The sequence FGL V/M PERP was formed at the junction of the deletion borders (S2 Fig). Due to incorporation of two proline residues in this sequence, the region was predicted to lose the α–helical structure, characteristic of the Eh1 motif [41]. This structural change, together with the loss of more than half of the conserved residues of the Eh1 motif, was predicted to inactivate Groucho/TLE recruitment activity in these FoxE1 proteins. Interestingly, the newly formed sequence was highly conserved across the analyzed placental mammals, which may indicate a potential functional significance. It is possible that the conserved sequence formed at the deletion junction site may confer a novel transcriptional function to these members of the FoxE subfamily.

### Analysis of non-B DNA structures forming in the echidna *FoxE1*

**Fig 2:**
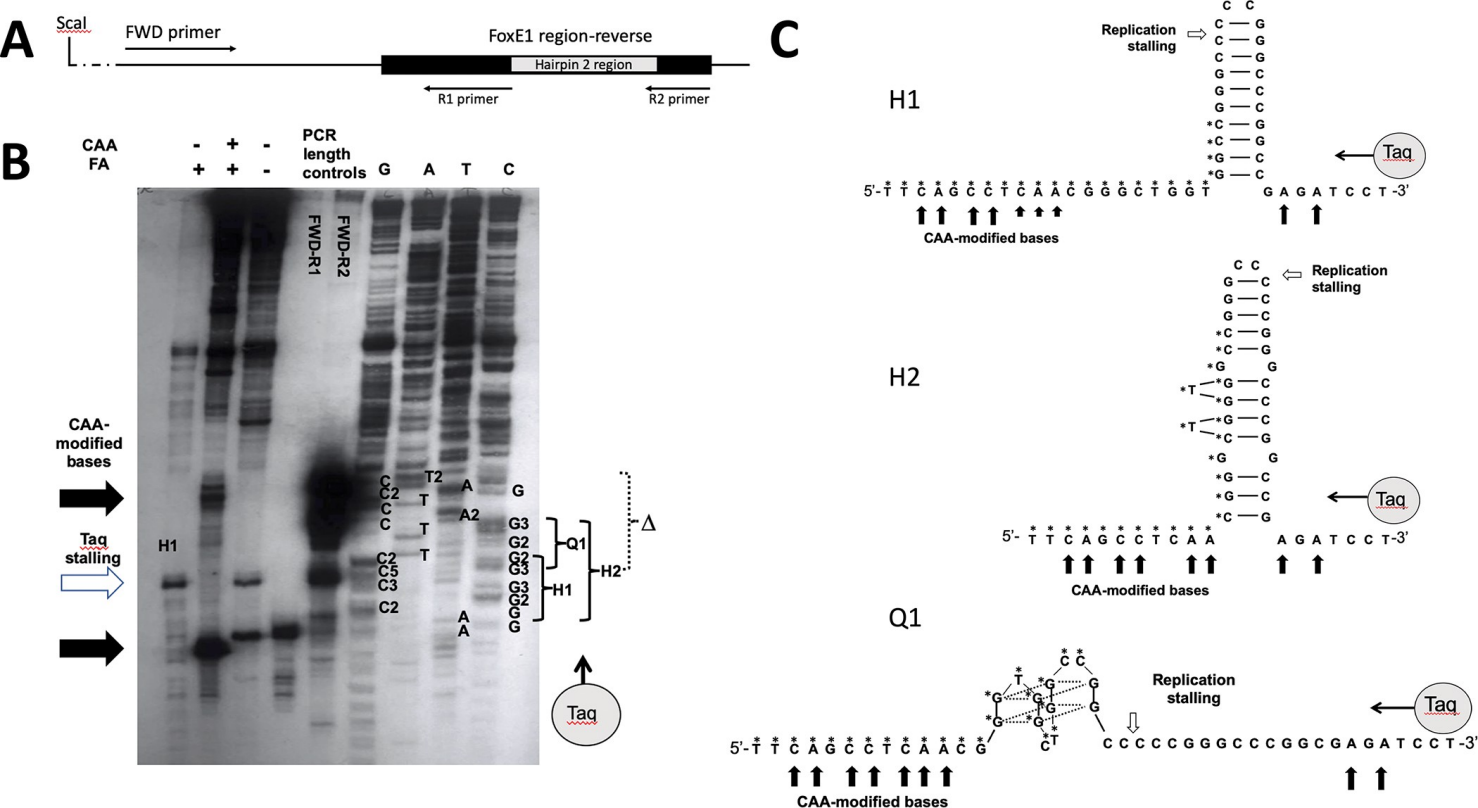
Analysis of secondary structures of the echidna *FoxE1* G-C rich region in PucNeoFoxRev plasmid with and without replication. A. Schematic of the PucNeoFoxRev area used in primer extension experiments. The position of FWD primer used in primer extension and R1 and R2 reverse primes used for the length controls are shown. B. The denaturing polyacrylamide gel electrophoresis of the primer extension reactions and control to analyze the structure of the deleted area of *FoxE1*. The first three lanes correspond to the primer extension reactions of the PucNeoFoxRev subjected to cellular ionic concentrations, then chloroacetaldehyde (CAA), linearized by ScaI digest, treated with formic acid (FA), and heated in TE to destroy the preexisting structures. Chemically modified DNA is in the second lane, while the first and the third are controls with omitted CAA or both CAA and FA. The regions that were modified are shown with black arrows on the left; the area of Taq polymerase stalling is shown with the white arrow. The following two lanes contain the length controls corresponding to the distances from the FWD primer to the start of the hairpin areas, or to the end of the *FoxE1* region, obtained by PCR with FWD and R1 or R2 primers. The last four lanes contain the Sanger sequencing reactions of PucNeoFoxRev plasmids performed with Thermo sequenase Cycle Sequencing Kit, based on Taq polymerase extension from FWD primer. The A, G, T, C lanes correspond to ddA, ddG, ddT, and ddA termination reactions of the synthesized strand; complementary nucleotides of the template strand are marked next to the termination bands. The following areas are shown in brackets next to the sequence: Δ, deleted area of *FoxE1*; H1, perfect hairpin; H2, extended imperfect hairpin; Q1, quadruplex area. The direction of Taq synthesis is from bottom to top (shown by a gray circle). C. The structures of hairpins H1 and H2, and quadruplex Q1 that can form in *FoxE1* deleted area.

To infer the mechanism of the Eh1 deletion, the area surrounding the deletion site was analyzed to assess the formation of non-B DNA structures. Non-B DNA structures, such as triplexes, hairpins, G-quartets, and Z-DNA, have been shown to be associated with genomic instability [21,22,24]. We examined the structure of the Eh1 deletion region using the *FoxE1* sequence of echidna (*Tachyglossus aculeatus)* (Fig 2A), which belongs to the order Monotremata that diversified right before placental mammals. The region overlapping and flanking the Eh1 encoding sequence of the echidna *FoxE1*gene is about 80% G-C rich. A portion of it was predicted to form a stable DNA hairpin by Mfold algorithm [42] (H1 hairpin, Fig 2C) [42]. Additionally, several G quartets were predicted by QGRS mapper [42]. However, in the G-rich regions, the formation of imperfect triplexes supported by G-C-G Hoogsteen triads [18] cannot be excluded.

**Fig 3:**
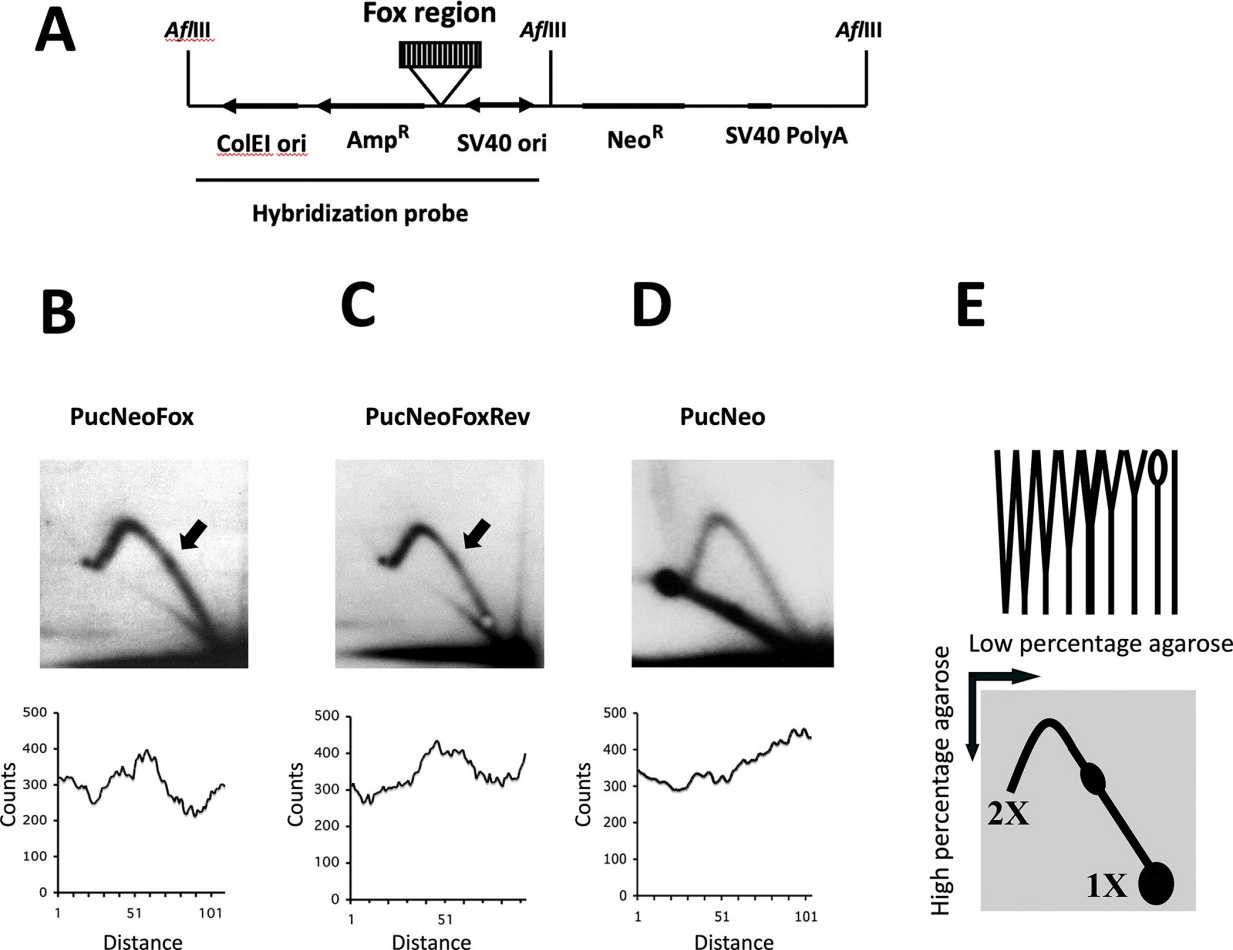
The echidna *FoxE1* hairpin-forming region causes replication stalling in COS-1 cells. (A) A map of PucNeoFox and PucNeoFoxRev plasmids. Hybridization probe and sites of AflIII digest used in two-dimensional electrophoresis assay are shown. (B-D) Two-dimensional agarose gel electrophoresis of replication intermediates of PucNeoFox (B), PucNeoFoxRev (C), and control (D) plasmids, digested with AflIII. The bulges on Y-arcs, corresponding to the replication stalling at the echidna *FoxE1* region are shown by arrows. Quantitative analyses of the middle areas of the Y-arcs containing the bulges are shown below. (E) A schematic representation of the two-dimensional gel electrophoresis. The top part shows the shapes of replication intermediates upon AflIII digest, ranging from 1X to 2X size; the bottom part represents their separation in two dimensions, resulting in the Y-arc. 1X spot consists of the molecules that are not currently replicating. A small bubble-shape intermediate is not visible on the 2D gel since it is covered by the 1X spot.

In order to explore the DNA structure at the deletion site, we performed an analysis of the single-stranded regions by chemical modifications [6]. Single-stranded DNA regions can be detected at the junctions of non-B structures with B DNA, and sometimes within DNA loops, which are often associated with non-B structures. To analyze the structures this DNA region can form, the deletion area of the echidna *FoxE1* was subcloned into pUCneo in two orientations (Figs 2A and 3A), and supercoiled plasmids were incubated at the ionic concentrations, typical for the intracellular environment. Then, the plasmids were exposed to chloroacetaldehyde, which modifies A and C nucleotides in single-stranded regions, but not within the double-stranded helix. Formic acid treatment was performed to break the DNA strands at the chloroacetaldehyde modification positions. The breaks corresponding to the DNA single-stranded areas were detected using a primer extension assay that shows terminations at the template breakpoints.

In this assay, we took extra care to distinguish the bands corresponding to the breaks at chloroacetaldehyde modification sites from the bands caused by Taq pausing at DNA secondary structures that can form prior or during primer extension reaction. We minimized the formation of those structures by linearizing the plasmids, and then heating at 65°C in low salt conditions with addition of EDTA. We also controlled for the structures that evaded this treatment, or still formed during primer extension, by performing the primer extension reaction on DNA that was not subjected to chloroacetaldehyde, but otherwise underwent the same procedure. Additionally, primer extensions were set with linearized templates that were preheated in TE to destroy pre-existing triplex structures, but have not been pre-incubated with ions or subjected to formic acid or chloroacetaldehyde treatments (conditions were indicated over the gel image in Fig 2B). Comparisons between samples helped understand if the bands resulted from inability of Taq to extend past the breaks or stalling by the structures that formed prior to or during the primer extension reactions.

The analysis indicated the presence of single-stranded areas at the borders of a potential extended hairpin H2 (Fig 2B, black arrows). Neither of the H1 or H2 hairpins predicted to form in this region could account for the observed Eh1 deletion since the endpoints for the deletion resided within the stem region of the hairpins (Fig 2C). However, the stalling point for the primer extension assay, which was not associated with hairpin formation (white arrow, Fig 2B), corresponded to the site of the deletion. Interestingly, the primer extension reaction stalled precisely at the border of the deletion, indicating that this position presented a roadblock for Taq replication (white arrow, Fig 2B). This stalling can be explained by a formation of the quadruplex structure Q1 (Fig 2C) right in front of the Taq polymerase; this quadruplex was predicted to be stable by QGRS mapper [42]. Structures resulting in stalled replication could promote cleavage of DNA, followed by a repair process that could result in a deletion [43]. The absence of primer extension breakpoints at this position in the sample subjected to chloroacetaldehyde suggested that Q1 structure was not forming without primer extension reaction, indicating that it only forms in a single-stranded DNA, which may exist temporarily during replication, but would not form within a double helix in non-replication conditions, even in the presence of supercoiling [44]. Interestingly, the chloroacetaldehyde modification pattern also suggested a quadruplex formation in the other orientation of *FoxE1* (Q2, S3 Fig). However, the area of this quadruplex did not overlap the deletion, nor did it affect primer extension, likely due to disassembly of this quadruplex structure under the assay conditions.

### The Eh1 region of the echidna *FoxE1* gene causes replication stalling in mammalian cells

To determine whether the potential hairpin region affects replication fork progression in vivo, the replication of pUCneoFox and pUCneoFoxR was examined in COS-1 monkey fibroblasts cells. These plasmids can support a bidirectional replication initiated at the SV40 origin [45], and the predicted *FoxE1* hairpin region was positioned in a manner to potentially interfere with the counterclockwise mammalian replication fork (Fig 3A). Replication progression through the *FoxE1* region was examined by two-dimensional separation of replication intermediates (Fig 3B–3D). Replication intermediates form a Y arc if the replication origin is located outside or close to the end of a fragment [46]. While the Y arc was smooth for the empty vector control plasmid (Fig 3D), replication of the plasmids containing the *FoxE1* insert in either orientation resulted in a Y-arc with a bulge (Fig 3B and 3C shown by an arrow). The bulges were located approximately one-third from the origin end, which corresponded to the position of the *FoxE1* insert. The Y-arc bulge represented an accumulation of replication intermediates at the position of *FoxE1* sequence, indicative of replication stalling in this area. The presence of the bulge was confirmed by quantitative analysis of the middle area of the arc (Fig 3B–3D bottom). While the two-dimensional analysis does not have sufficient resolution to define the structure that causes replication stalling, it is likely that the same structure is responsible for both the endpoint of primer extension in vitro and the replication fork stalling in cells.

## Discussion

This study focused on a functional change in Fox family proteins that occurred during the evolutionary divergence of placental mammals: the loss of the Engrailed Homology 1 motif. Comparative sequence analysis demonstrated that the Eh1 motif was lost from FoxE1 proteins of all placental mammals, with the evolutionary timing of the loss roughly coinciding with the diversification of the placental mammalian lineage. Since the Eh1 motif mediates the recruitment of Groucho/TLE co-repressors [47], the loss of the functional Eh1 motif likely resulted in a loss of the Groucho/TLE-dependent repressive function for these FoxE1 proteins. We also identified a new conserved motif formed in FoxE1 at the junction of the deletion borders, which could potentially confer a new transcriptional function. Consistent with this suggestion of a change in FoxE1 function, FoxE1 is required for the chondrogenesis of the zebrafish pharyngeal skeleton [48], but not for the thyroid development, as has been observed for the FoxE1 of placental mammals [15].

A loss of the Eh1 motif has been observed for other transcriptional factor families. Two homeodomain-containing proteins, Msx2 and Msx3, lost the C-terminal Eh1 motifs following duplication in the vertebrate lineage. In contrast, a vertebrate Msx1 protein and an ancestral form of Msx protein found in amphioxus, contain both N-terminal and C-terminal Eh1 motifs [49]. Moreover, both motifs are functional in the Msx protein, and mediate binding to the Groucho-related co-repressor, Grg1 [50]. In our previous study, we have also reported a similar loss of the Eh1 motif in *FoxE1* genes in the avian lineage [51]. We identified two microdeletions that resulted in a loss of the Eh1 motif and another repressive domain, which indicated a significant divergence of the avian FoxE1 proteins from their amphibian and mammalian versions.

The modular structure of transcriptional regulatory proteins provides an efficient mechanism for the modification of protein structure and function during evolution. Gain and loss of cofactor interaction motifs in trans-regulatory domains has been linked to acquisition of novel regulatory functions [52]. An example of a loss and a gain of cofactor interaction motifs was observed in an orthologous group of the pair rule transcription factor, Fushi tarazu (Ftz). The F/YPWM motif of the *Schistocerca* Ftz protein is required for the homeotic function and interaction with the transcriptional cofactor, extradenticle/Pdx. This motif is also present andappears to be necessary for a similar homeotic function in the ancestral form of the Ftz protein in *Crustaceans* [53]. However, the *Drosophila* Ftz protein has lost this motif and the associated homeotic function, while acquiring a novel embryonic function in regulation of segmentation [54]. This newly acquired segmentation function of *Drosophila* Ftz correlated with acquisition of a novel cofactor interaction motif, LXXLL [55]. This short motif has been shown to be involved in the interaction of the *Drosophila* Ftz protein with the nuclear receptor F1-Ftz during segmentation [56,57].

In this study, we investigated the deletion mechanism that led to the loss of the Eh1 motif in *FoxE1* genes. The deleted area is within a highly G-C rich region that can potentially adopt a number of non-B conformations. Analysis of sequences surrounding the deletion site predicted the potential to form perfect or extended imperfect hairpins, as well as quadruplexes. However, it is unclear which specific structures form in the sequence, and especially which structure formation is responsible for the observed microdeletion of the Eh1 motif. The primer extension analysis indicated that the deletion border corresponded precisely with the site of replication stalling, consistent with the formation of a quadruplex structure at this position.

Quadruplexes (G quartets or G4 DNA structures) are stabilized by Hoogsteen bonds between the tetrads of G nucleotides. Additionally, the adjacent tetrads are also involved in π-π interactions, so called stacking [58]. Formation of quadruplexes requires the presence of four clusters of at least two adjacent Gs that form tetrads, separated by not more than 7 nucleotides that form the loops [59]. The stability of quadruplexes depends on many parameters, including the composition and length of the loops and the composition of the adjacent regions [60]. Quadruplexes were shown to interfere with DNA replication, in both *in vitro* and *in vivo* assays [61,62]; however, there are natural mechanisms in the cell that unwind quadruplex structures to allow the replication to proceed [63]. The observed deletion is consistent with a proposed mechanism of Dna2 endonuclease cleavage in a single-stranded loop of the quadruplex [64], followed by excision of the structure by EXO1 exonuclease [64]. Interestingly, the hairpin structure that formed independently of replication (detected by chloroacetaldehyde modification of non-replicating plasmid DNA), did not correspond to the deletion borders, eliminating this structure as the underlying cause of the deletion. This observation is consistent with previous studies showing that not all predicted quadruplexes would readily form in the supercoiled DNA, but require a single strand formation as a result of transcription or replication [44].

Our in vivo studies in mammalian cells confirmed the obstructed replication within the same region of the echidna *FoxE1*. However, the two-dimensional analysis of replication intermediates does not provide a single-nucleotide resolution needed to determine the exact position of replication stalling. We and others have previously reported replication stalling at a triplex-forming sequence, GAA repeat [33,65,66], similar to the one we observed here [66]. We have also previously shown that d(G)n·d(C)n repeats block DNA replication progression in yeast [67], while this effect was further enhanced by stalled transcription caused by R-loops, stable complexes between DNA and RNA [68].

The sequence that the Taq polymerase stalled at is a perfect candidate for a quadruplex, however, it cannot be excluded that the same sequence can also adopt an imperfect triplex, based on its high G-C content. Recent studies demonstrated a major role of triplex structures in genome instability [40,69]. Using the method of S1-END-seq, thousands of single-stranded regions were detected at sequences predicted to form triplexes [40]. The formation of these structures was more pronounced in cycling cells and correlated with replication. The triplex structures were also shown to be hotspots of genomic instability in several studies. Intriguingly, the formation of triplex structures was shown to increase during differentiation of iPSCs, which may indicate that conditions existing during specific developmental windows can favor triplex formation and associated genomic instability [40]. However, the formation of such structures may occur with low probability, and further selection would determine whether the triplex-associated mutations or deletions lead to development of beneficial properties.

A similar example of a loss of a DNA region affecting evolution has been demonstrated for the Stickleback fish that adapted to freshwater environment. The adaptation was associated with a deletion of an enhancer located inside an area prone to the formation of an alternative DNA structure, presumably Z-DNA forming within a long TG-repeat. The deletion area was also shown to cause replication-dependent fragility, which can explain the elevated mutation rate that was likely responsible for this evolutionary change [70].

Our results support the conclusion that in replication or non-replication conditions, different structures can form in the *FoxE1* deletion region. In the context of non-replicating double-stranded supercoiled DNA, the deleted area displays a chloroacetaldehyde modification pattern likely corresponding to a hairpin formation, while in replication conditions, the position of replication stalling was consistent with the formation of a quadruplex. The quadruplex hypothesis is more consistent with the *FoxE1* deletion borders. These insights into the cause and consequences of the loss of Eh1 motif in Fox family may promote a better understanding of the genomic drivers of the evolution of placental mammals.

## Supporting information

S1 Fig Multiple sequence alignment of the C-terminal segment of FoxE1 proteins from 22 vertebrate species. The color represents the scoring of the alignment. Multiple sequence alignments were constructed using T-COFFEE, version 7.7.1 [3].
(TIFF)

S2 Fig A consensus of an evolutionary novel sequence formed as a result of the microdeletion of the Eh1 motif and adjacent region in the Fox proteins of placental mammals. The diagram was generated with the WebLogo program [71].
(TIFF)

S3 Fig Analysis of secondary structures of the echidna FoxE1 G-C rich region in PucNeoFox plasmid with and without replication. A. Schematic of the PucNeoFox area used in primer extension experiments. The position of FWD primer used in primer extension is shown. B. The denaturing polyacrylamide gel electrophoresis of the primer extension reactions and control to analyze the structure of the deleted area of *FoxE1*. The first three lanes correspond to the primer extension reactions of the PucNeoFox subjected to cellular ionic concentrations, then chloroacetaldehyde (CAA), linearized by ScaI digest, treated with formic acid (FA), and heated in TE to destroy the preexisting structures. Chemically modified DNA is in the second lane, while the first and the third are controls with omitted CAA or both CAA and FA. The regions that were modified are shown with black arrows on the left; the area of Taq polymerase stalling is at the structure forming at the border of *FoxE1* and plasmid regions, hence not indicated by the arrow. The last four lanes contain the Sanger sequencing reactions of PucNeoFox plasmids performed with Thermo sequenase cycling kit, based on Taq polymerase extension from FWD primer. The A, G, T, C lanes correspond to ddA, ddG, ddT, and ddA termination reactions of the synthesized strand; complementary nucleotides of the template strand are marked next to the termination bands. The following areas are shown in brackets next to the sequence: Δ, deleted area of FoxE1; Q2, quadruplex area. The direction of Taq synthesis is from bottom to top (shown by a gray circle). C. The structures of quadruplex Q2 that can form adjacent to the *FoxE1* deletion region.
(TIFF)

S1 Raw image (TIFF)

## References

[1] Yaklichkin S, Vekker A, Stayrook S, Lewis M, Kessler DS. Prevalence of the EH1 Groucho interaction motif in the metazoan Fox family of transcriptional regulators. BMC Genomics. 2007;8. doi: 10.1186/1471-2164-8-201 17598915 PMC1939712

[2] Higgins DG, Thompson JD, Gibson TJ. [22] Using CLUSTAL for multiple sequence alignments. Methods Enzymol. 1996;266. doi: 10.1016/s0076-6879(96)66024-8 8743695

[3] Notredame C, Higgins DG, Heringa J. T-coffee: A novel method for fast and accurate multiple sequence alignment. J Mol Biol. 2000;302(1). doi: 10.1006/jmbi.2000.4042 10964570

[4] Chandok GS, Kapoor KK, Brick RM, Sidorova JM, Krasilnikova MM. A distinct first replication cycle of DNA introduced in mammalian cells. Nucleic Acids Res. 2011;39(6). doi: 10.1093/nar/gkq903 21062817 PMC3064806

[5] Krasilnikova MM, Mirkin SM. Replication Stalling at Friedreich’s Ataxia (GAA)n Repeats In Vivo. Mol Cell Biol. 2004;24(6). doi: 10.1128/MCB.24.6.2286-2295.2004 14993268 PMC355872

[6] Kohwi Y, Kohwi-Shigematsu T. Magnesium ion-dependent triple-helix structure formed by homopurine-homopyrimidine sequences in supercoiled plasmid DNA. Proc Natl Acad Sci U S A. 1988;85(11). doi: 10.1073/pnas.85.11.3781 3375241 PMC280302

[7] Hirt B. Selective extraction of polyoma DNA from infected mouse cell cultures. J Mol Biol. 1967;26(2). doi: 10.1016/0022-2836(67)90307-5 4291934

[8] Krasilnikova MM, Mirkin SM. Analysis of triplet repeat replication by two-dimensional gel electrophoresis. Methods Mol Biol. 2004;277. doi: 10.1385/1-59259-804-8:019 15201446

[9] Wang JC, Waltner-Law M, Yamada K, Osawa H, Stifani S, Granner DK. Transducin-like enhancer of split proteins, the human homologs of Drosophila Groucho, interact with hepatic nuclear factor 3β. Journal of Biological Chemistry. 2000;275(24). 10.1074/jbc.M910211199 10748198

[10] Andrioli LP, Oberstein AL, Corado MSG, Yu D, Small S. Groucho-dependent repression by Sloppy-paired 1 differentially positions anterior pair-rule stripes in the Drosophila embryo. Dev Biol. 2004;276(2). doi: 10.1016/j.ydbio.2004.09.025 15581884

[11] Roth M, Bonev B, Lindsay J, Lea R, Panagiotaki N, Houart C, et al. FoxG1 and TLE2 act cooperatively to regulate ventral telencephalon formation. Development. 2010;137(9). doi: 10.1242/dev.044909 20356955 PMC2853852

[12] Reid CD, Steiner AB, Yaklichkin S, Lu Q, Wang S, Hennessy M, et al. FoxH1 mediates a Grg4 and Smad2 dependent transcriptional switch in Nodal signaling during Xenopus mesoderm development. Dev Biol. 2016;414(1). doi: 10.1016/j.ydbio.2016.04.006 27085753 PMC4875808

[13] Charney RM, Forouzmand E, Cho JS, Cheung J, Paraiso KD, Yasuoka Y, et al. Foxh1 Occupies cis-Regulatory Modules Prior to Dynamic Transcription Factor Interactions Controlling the Mesendoderm Gene Program. Dev Cell. 2017;40(6). doi: 10.1016/j.devcel.2017.02.017 28325473 PMC5434453

[14] Sekiya T, Zaret KS. Repression by Groucho/TLE/Grg Proteins: Genomic Site Recruitment Generates Compacted Chromatin In Vitro and Impairs Activator Binding In Vivo. Mol Cell. 2007;28(2). doi: 10.1016/j.molcel.2007.10.002 17964267 PMC2083644

[15] de Felice M, Ovitt C, Biffali E, Rodriguez-Mallon A, Arra C, Anastassiadis K, et al. A mouse model for hereditary thyroid dysgenesis and cleft palate. Nat Genet. 1998;19(4). doi: 10.1038/1289 9697704

[16] Castanet M, Park SM, Smith A, Bost M, Léger J, Lyonnet S, et al. A novel loss-of-function mutation in TTF-2 is associated with congenital hypothyroidism, thyroid agenesis and cleft palate. Hum Mol Genet. 2002;11(17). doi: 10.1093/hmg/11.17.2051 12165566

[17] Clifton-Bligh RJ, Wentworth JM, Heinz P, Crisp MS, John R, Lazarus JH, et al. Mutation of the gene encoding human TTF-2 associated with thyroid agenesis, cleft palate and choanal atresia. Nat Genet. 1998;19(4). doi: 10.1038/1294 9697705

[18] Frank-Kamenetskii MD, Mirkin SM. Triplex DNA structures. Vol. 64, Annual Review of Biochemistry. 1995. doi: 10.1146/annurev.bi.64.070195.000433 7574496

[19] Mirkin SM. Discovery of alternative DNA structures: A heroic decade (1979–1989). Vol. 13, Frontiers in Bioscience. 2008. doi: 10.2741/2744 17981612

[20] Khristich AN, Mirkin SM. On the wrong DNA track: Molecular mechanisms of repeat-mediated genome instability. Vol. 295, Journal of Biological Chemistry. 2020. doi: 10.1074/jbc.REV119.007678 32060097 PMC7105313

[21] McKinney JA, Wang G, Mukherjee A, Christensen L, Subramanian SHS, Zhao J, et al. Distinct DNA repair pathways cause genomic instability at alternative DNA structures. Nat Commun. 2020;11(1). doi: 10.1038/s41467-019-13878-9 31932649 PMC6957503

[22] Georgakopoulos-Soares I, Morganella S, Jain N, Hemberg M, Nik-Zainal S. Noncanonical secondary structures arising from non-B DNA motifs are determinants of mutagenesis. Genome Res. 2018;28(9). doi: 10.1101/gr.231688.117 30104284 PMC6120622

[23] Guiblet WM, DeGiorgio M, Cheng X, Chiaromonte F, Eckert KA, Huang YF, et al. Selection and thermostability suggest G-quadruplexes are novel functional elements of the human genome. Genome Res. 2021;31(7). doi: 10.1101/gr.269589.120 34187812 PMC8256861

[24] Zhao J, Wang G, del Mundo IM, McKinney JA, Lu X, Bacolla A, et al. Distinct Mechanisms of Nuclease-Directed DNA-Structure-Induced Genetic Instability in Cancer Genomes. Cell Rep. 2018;22(5). doi: 10.1016/j.celrep.2018.01.014 29386108 PMC6011834

[25] Maizels N, Gray LT. The G4 Genome. PLoS Genet. 2013;9(4). doi: 10.1371/journal.pgen.1003468 23637633 PMC3630100

[26] Sinden RR, Pytlos-Sinden MJ, Potaman VN. Slipped strand DNA structures. Vol. 12, Frontiers in Bioscience. 2007. doi: 10.2741/2427 17569609

[27] Pelletier R, Krasilnikova MM, Samadashwily GM, Lahue R, Mirkin SM. Replication and Expansion of Trinucleotide Repeats in Yeast. Mol Cell Biol. 2003;23(4). doi: 10.1128/MCB.23.4.1349-1357.2003 12556494 PMC141142

[28] Zhang H, Freudenreich CH. An AT-Rich Sequence in Human Common Fragile Site FRA16D Causes Fork Stalling and Chromosome Breakage in S. cerevisiae. Mol Cell. 2007;27(3). doi: 10.1016/j.molcel.2007.06.012 17679088 PMC2144737

[29] Voineagu I, Narayanan V, Lobachev KS, Mirkin SM. Replication stalling at unstable inverted repeats: Interplay between DNA hairpins and fork stabilizing proteins. Proc Natl Acad Sci U S A. 2008;105(29). doi: 10.1073/pnas.0804510105 18632578 PMC2481305

[30] Voineagu I, Surka CF, Shishkin AA, Krasilnikova MM, Mirkin SM. Replisome stalling and stabilization at CGG repeats, which are responsible for chromosomal fragility. Nat Struct Mol Biol. 2009;16(2). doi: 10.1038/nsmb.1527 19136957 PMC2837601

[31] Matos-Rodrigues G, Hisey JA, Nussenzweig A, Mirkin SM. Detection of alternative DNA structures and its implications for human disease. Mol Cell. 2023; doi: 10.1016/j.molcel.2023.08.018 37863029 PMC12673489

[32] Lee WTC, Yin Y, Morten MJ, Tonzi P, Gwo PP, Odermatt DC, et al. Single-molecule imaging reveals replication fork coupled formation of G-quadruplex structures hinders local replication stress signaling. Nat Commun. 2021;12 (1). doi: 10.1038/s41467-021-22830-9 33953191 PMC8099879

[33] Follonier C, Oehler J, Herrador R, Lopes M. Friedreich’s ataxia-associated GAA repeats induce replication-fork reversal and unusual molecular junctions. Nat Struct Mol Biol. 2013;20(4). doi: 10.1038/nsmb.2520 23454978

[34] Freudenreich CH. Chromosome fragility: Molecular mechanisms and cellular consequences. Vol. 12, Frontiers in Bioscience. 2007. doi: 10.2741/2437 17569619

[35] Lobachev KS, Stenger JE, Kozyreva OG, Jurka J, Gordenin DA, Resnick MA. Inverted Alu repeats unstable in yeast are excluded from the human genome. EMBO Journal. 2000;19(14). doi: 10.1093/emboj/19.14.3822 10899135 PMC313988

[36] Wells RD. Discovery of the role of non-B DNA structures in mutagenesis and human genomic disorders. J Biol Chem. 2009;284(14). doi: 10.1074/jbc.X800010200 19054760 PMC2666547

[37] Lobachev KS, Shor BM, Tran HT, Taylor W, Keen JD, Resnick MA, et al. Factors affecting inverted repeat stimulation of recombination and deletion in Saccharomyces cerevisiae. Genetics. 1998;148(4). doi: 10.1093/genetics/148.4.1507 9560370 PMC1460095

[38] Chan YF, Marks ME, Jones FC, Villarreal G, Shapiro MD, Brady SD, et al. Adaptive evolution of pelvic reduction in sticklebacks by recurrent deletion of a pitxl enhancer. Science (1979). 2010;327(5963). 10.1126/science.1182213 PMC3109066 20007865

[39] Kelkar YD, Tyekucheva S, Chiaromonte F, Makova KD. The genome-wide determinants of human and chimpanzee microsatellite evolution. Genome Res. 2008;18(1). doi: 10.1101/gr.7113408 18032720 PMC2134767

[40] Matos-Rodrigues G, van Wietmarschen N, Wu W, Tripathi V, Koussa NC, Pavani R, et al. S1-END-seq reveals DNA secondary structures in human cells. Mol Cell. 2022 Oct 6;82(19):3538–52. doi: 10.1016/j.molcel.2022.08.007 36075220 PMC9547894

[41] Jennings BH, Pickles LM, Wainwright SM, Roe SM, Pearl LH, Ish-Horowicz D. Molecular Recognition of Transcriptional Repressor Motifs by the WD Domain of the Groucho/TLE Corepressor. Mol Cell. 2006;22(5). doi: 10.1016/j.molcel.2006.04.024 16762837

[42] Kikin O D’Antonio L, Bagga PS. QGRS Mapper: A web-based server for predicting G-quadruplexes in nucleotide sequences. Nucleic Acids Res. 2006;34(WEB. SERV. ISS.). 10.1093/nar/gkl253 PMC1538864 16845096

[43] Schmidt MHM, Pearson CE. Disease-associated repeat instability and mismatch repair. Vol. 38, DNA Repair. 2016. doi: 10.1016/j.dnarep.2015.11.008 26774442

[44] Sekibo DAT, Fox KR. The effects of DNA supercoiling on G-quadruplex formation. Nucleic Acids Res. 2017;45(21). doi: 10.1093/nar/gkx856 29036619 PMC5716088

[45] Bergsma DJ, Olive DM, Hartzell SW, Subramanian KN. Territorial limits and functional anatomy of the simian virus 40 replication origin. Proc Natl Acad Sci U S A. 1982;79(2 I). doi: 10.1073/pnas.79.2.381 6281769 PMC345744

[46] Friedman KL, Brewer BJ. Analysis of Replication Intermediates by Two-Dimensional Agarose Gel Electrophoresis. Methods Enzymol. 1995;262(C). doi: 10.1016/0076-6879(95)62048-6 8594382

[47] Yaklichkin S, Steiner AB, Lu Q, Kessler DS. FoxD3 and Grg4 physically interact to repress transcription and induce mesoderm in Xenopus. Journal of Biological Chemistry. 2007;282(4). doi: 10.1074/jbc.M607412200 17138566 PMC1780074

[48] Nakada C, Iida A, Tabata Y, Watanabe S. Forkhead transcription factor foxe1 regulates chondrogenesis in zebrafish. J Exp Zool B Mol Dev Evol. 2009;312(8). doi: 10.1002/jez.b.21298 19488987

[49] Finnerty JR, Mazza ME, Jezewski PA. Domain duplication, divergence, and loss events in vertebrate Msx paralogs reveal phylogenomically informed disease markers. BMC Evol Biol. 2009;9(1). doi: 10.1186/1471-2148-9-18 19154605 PMC2655272

[50] Takahashi H, Kamiya A, Ishiguro A, Suzuki AC, Saitou N, Toyoda A, et al. Conservation and diversification of Msx protein in metazoan evolution. Mol Biol Evol. 2008;25(1). doi: 10.1093/molbev/msm228 17940209

[51] Yaklichkin SY, Darnell DK, Pier M v., Antin PB, Hannenhalli S. Accelerated evolution of 3’avian FOXE1 genes, and thyroid and feather specific expression of chicken FoxE1. BMC Evol Biol. 2011;11(1). 10.1186/1471-2148-11-302 PMC3207924 21999483

[52] Hsia CC, McGinnis W. Evolution of transcription factor function. Vol. 13, Current Opinion in Genetics and Development. 2003. doi: 10.1016/s0959-437x(03)00017-0 12672498

[53] Mouchel-Vielh E, Blin M, Rigolot C, Deutsch JS. Expression of a homologue of the fushi tarazu (ftz) gene in a cirripede crustacean. Evol Dev. 2002;4 (2). 10.1046/j.1525-142x.2002.01063.x 12004965

[54] Löhr U, Pick L. Cofactor-interaction motifs and the cooption of a homeotic Hox protein into the segmentation pathway of Drosophila melanogaster. Current Biology. 2005;15(7). doi: 10.1016/j.cub.2005.02.048 15823536

[55] Guichet A, Copeland JWR, Erdélyi M, Hlousek D, Závorszky P, Ho J, et al. The nuclear receptor homologue Ftz-F1 and the homeodomain protein Ftz are mutually dependent cofactors. Nature. 1997;385(6616). doi: 10.1038/385548a0 9020363

[56] Mouchel-Vielh E, Rigolot C, Gibert JM, Deutsch JS. Molecules and the Body Plan: TheHoxGenes of Cirripedes (Crustacea). Mol Phylogenet Evol. 1998 Jun 1;9(3):382–9. doi: 10.1006/mpev.1998.0498 9667986

[57] Yussa M, Löhr U, Su K, Pick L. The nuclear receptor Ftz-F1 and homeodomain protein Ftz interact through evolutionarily conserved protein domains. Mech Dev. 2001;107(1–2). doi: 10.1016/s0925-4773(01)00448-8 11520662

[58] Gellert M, Lipsett MN, Davies DR. Helix formation by guanylic acid. Proc Natl Acad Sci U S A. 1962;48. doi: 10.1073/pnas.48.12.2013 13947099 PMC221115

[59] Lerner LK, Sale JE. Replication of G quadruplex DNA. Vol. 10, Genes. 2019. doi: 10.3390/genes10020095 30700033 PMC6409989

[60] Jana J, Weisz K. Thermodynamic Stability of G-Quadruplexes: Impact of Sequence and Environment. Vol. 22, ChemBioChem. 2021. doi: 10.1002/cbic.202100127 33844423 PMC8518667

[61] Weitzmann MN, Woodford KJ, Usdin K. DNA secondary structures and the evolution of hypervariable tandem arrays. Journal of Biological Chemistry. 1997;272(14). doi: 10.1074/jbc.272.14.9517 9083093

[62] Sarkies P, Reams C, Simpson LJ, Sale JE. Epigenetic Instability due to Defective Replication of Structured DNA. Mol Cell. 2010;40(5). doi: 10.1016/j.molcel.2010.11.009 21145480 PMC3145961

[63] Sato K, Martin-Pintado N, Post H, Altelaar M, Knipscheer P. Multistep mechanism of G-quadruplex resolution during DNA replication. Sci Adv. 2021;7(39). doi: 10.1126/sciadv.abf8653 34559566 PMC8462899

[64] Stroik S, Kurtz K, Lin K, Karachenets S, Myers CL, Bielinsky AK, et al. EXO1 resection at G-quadruplex structures facilitates resolution and replication. Nucleic Acids Res. 2020;48(9). doi: 10.1093/nar/gkaa199 32232411 PMC7229832

[65] Chandok GS, Patel MP, Mirkin SM, Krasilnikova MM. Effects of Friedreich’s ataxia GAA repeats on DNA replication in mammalian cells. Nucleic Acids Res. 2012;40(9). doi: 10.1093/nar/gks021 22262734 PMC3351192

[66] Khristich AN, Armenia JF, Matera RM, Kolchinski AA, Mirkin SM. Large-scale contractions of Friedreich’s ataxia GAA repeats in yeast occur during DNA replication due to their triplex-forming ability. Proc Natl Acad Sci U S A. 2020;117(3). doi: 10.1073/pnas.1913416117 31911468 PMC6983365

[67] Krasilnikova MM M. samadashwily G, Krasilnikov AS, Mirkin SM. Transcription through a simple DNA repeat blocks replication elongation. EMBO Journal. 1998;17(17). 10.1093/emboj/17.17.5095 PMC1170837 9724645

[68] Belotserkovskii BP, Liu R, Tornaletti S, Krasilnikova MM, Mirkin SM, Hanawalt PC. Mechanisms and implications of transcription blockage by guanine-rich DNA sequences. Proc Natl Acad Sci U S A. 2010;107(29). doi: 10.1073/pnas.1007580107 20616059 PMC2919923

[69] Maekawa K, Yamada S, Sharma R, Chaudhuri J, Keeney S. Triple-helix potential of the mouse genome. Proc Natl Acad Sci U S A. 2022;119(19). doi: 10.1073/pnas.2203967119 35503911 PMC9171763

[70] Xie KT, Wang G, Thompson AC, Wucherpfennig JI, Reimchen TE, MacColl ADC, et al. DNA fragility in the parallel evolution of pelvic reduction in stickleback fish. Science (1979). 2019;363(6422). doi: 10.1126/science.aan1425 30606845 PMC6677656

[71] Crooks GE, Hon G, Chandonia JM, Brenner SE. WebLogo: A sequence logo generator. Genome Res. 2004;14(6). doi: 10.1101/gr.849004 15173120 PMC419797

