# Supplementary figures and images for: The evolutionary loss of the Eh1 motif in FoxE1 in the lineage of placental mammals
Source: PLoS One. 2023 Dec 27;18(12):e0296176. doi: 10.1371/journal.pone.0296176 (PMC10752562; doi:10.1371/journal.pone.0296176)

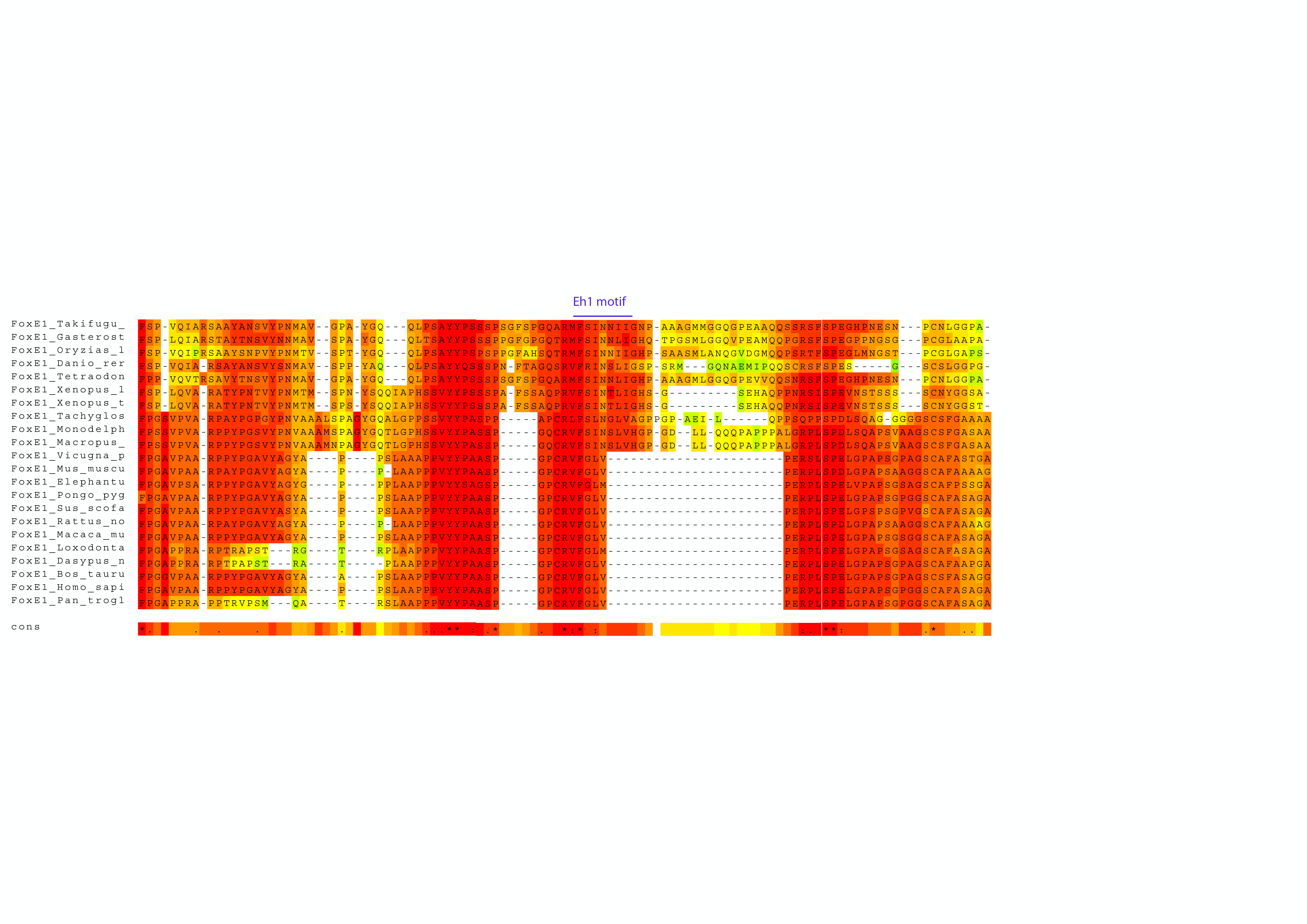

Supplement: S1 Fig — The color represents the scoring of the alignment. Multiple sequence alignments were constructed using T-COFFEE, version 7.7.1 [3]. (TIFF) [file pone.0296176.s001.tiff]

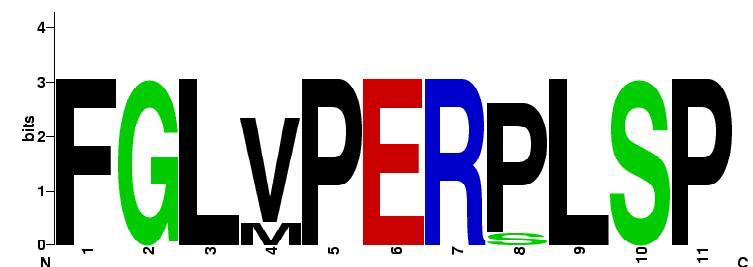

Supplement: S2 Fig — The diagram was generated with the WebLogo program [71]. (TIFF) [file pone.0296176.s002.tiff]

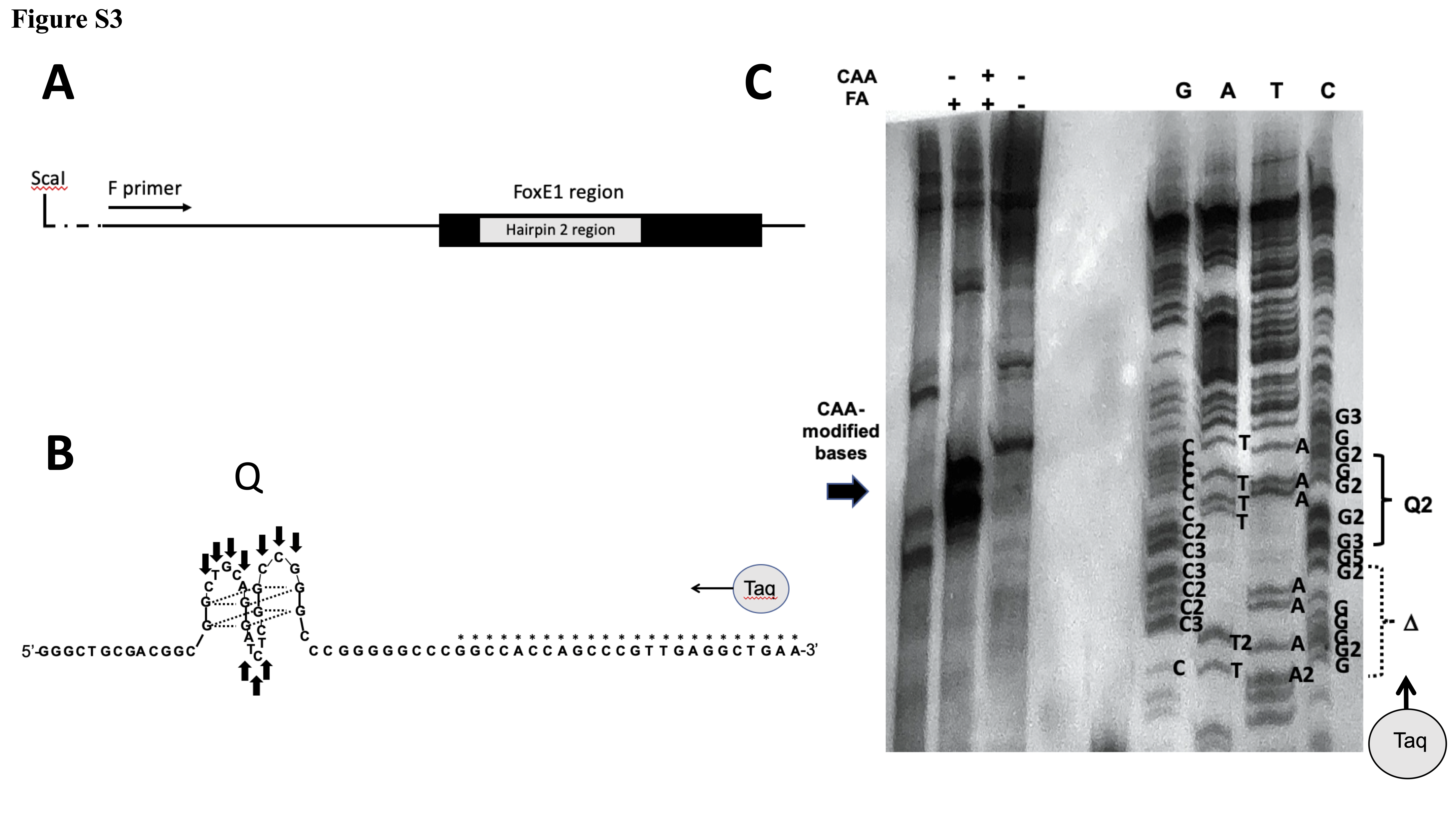

Supplement: S3 Fig — A. Schematic of the PucNeoFox area used in primer extension experiments. The position of FWD primer used in primer extension is shown. B. The denaturing polyacrylamide gel electrophoresis of the primer extension reactions and control to analyze the structure of the deleted area of FoxE1. The first three lanes correspond to the primer extension reactions of the PucNeoFox subjected to cellular ionic concentrations, then chloroacetaldehyde (CAA), linearized by ScaI digest, treated with formic acid (FA), and heated in TE to destroy the preexisting structures. Chemically modified DNA is in the second lane, while the first and the third are controls with omitted CAA or both CAA and FA. The regions that were modified are shown with black arrows on the left; the area of Taq polymerase stalling is at the structure forming at the border of FoxE1 and plasmid regions, hence not indicated by the arrow. The last four lanes contain the Sanger sequencing reactions of PucNeoFox plasmids performed with Thermo sequenase cycling kit, based on Taq polymerase extension from FWD primer. The A, G, T, C lanes correspond to ddA, ddG, ddT, and ddA termination reactions of the synthesized strand; complementary nucleotides of the template strand are marked next to the termination bands. The following areas are shown in brackets next to the sequence: Δ, deleted area of FoxE1; Q2, quadruplex area. The direction of Taq synthesis is from bottom to top (shown by a gray circle). C. The structures of quadruplex Q2 that can form adjacent to the FoxE1 deletion region. (TIFF) [file pone.0296176.s003.tiff]

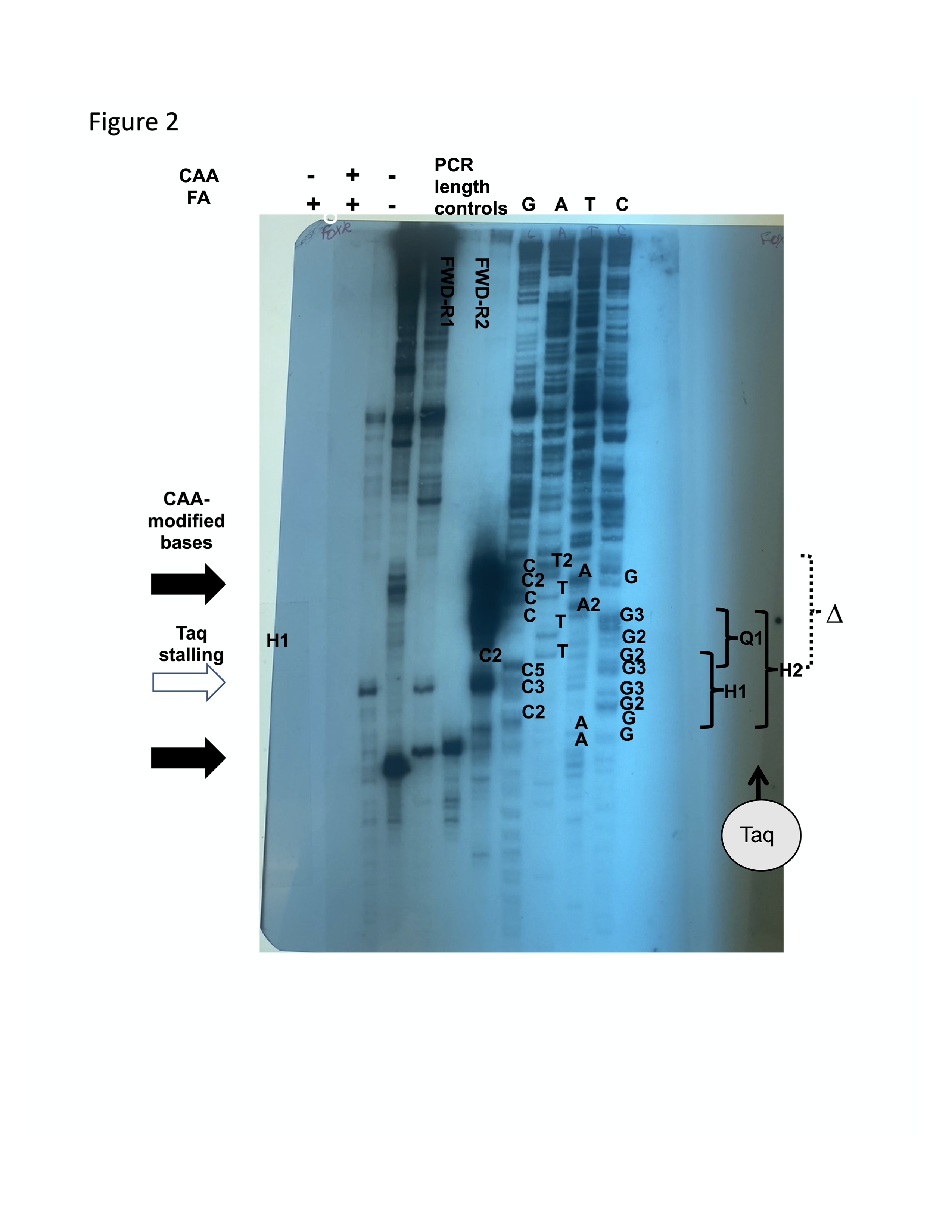

Supplement: S1 Raw image — (TIFF) [file pone.0296176.s004.tiff]
